# A New and Expeditious Mode of Budding

**Published:** 1811-03

**Authors:** Thomas Andrew Knight


### *A new and expeditious Mode of Budding.* By Thomas Andrew Knight, Esq. F. R. S.

[Transactions of the Horticultural Society of London.]

Parkinson, in his Paradisus Londoniensis, which was published in 1629, has observed, that the nurserymen of his days had been so long in the practice of substituting one variety of fruit for another, that the habit of doing so was almost become hereditary amongst them: were we to judge from the modern practice, in some public nurseries, we might suspect the possessors of them to be the offspring of intermarriages between the descendants of those alluded to by Parkinson. He has, however, mentioned his "very good friend, Master John Tradescant" and "Master John Miller," as exceptions; and similar exceptions are, I believe, to be found in modern days. It must, however, be admitted, that wherever the character of the leaf does not expose the error of the grafter, as in the different varieties of the peach and nectarine, mistakes will sometimes occur; and therefore a mode of changing the variety, or of introducing a branch of another variety, with great expedition, may possibly be acceptable to many readers of the Horticultural Transactions.

The

The luxuriant shoots of peach and nectarine trees are generally barren; but the lateral shoots emitted in the same season by them are often productive of fruit, particularly if treated in the manner recommended by me in the Horticultural Transactions of 1808, p. 88. In the experiments I have there described, the bearing wood was afforded by the natural buds of the luxuriant shoots: but I thought it probable that such might as readily be afforded by the inserted buds of another variety, under appropriate management. I therefore, as early in the month of June, of the year 1808, as the luxuriant shoots of my peach trees were grown sufficiently firm to permit the operation, inserted buds of other varieties into them, employing two distinct ligatures to hold the buds in their places. One ligature was first placed above the bud inserted; and upon the transverse section through the bark: the other which had no farther office than that of securing the bud, was applied in the usual way. As soon as the buds (which never fail under the preceding circumstances) had attached themselves, the ligatures last applied were taken off, but the others were suffered to remain. The passage of the sap upwards was in consequence much obstructed, and the inserted buds began to vegetate strongly in July: and when these had afforded shoots about four inches long, the remaining ligatures were taken off, to permit the excess of sap to pass on; and the young shoots were nailed to the wall. Being there properly exposed to light, their wood ripened well, and afforded blossoms in the succeeding spring: this would, I do not doubt, have afforded fruit; but that, leaving my residence at Elton for this place, I removed my trees, and the whole of their blossoms in the last spring proved, in consequence, equally abortive.

